# Transnasal Endoscopic Pituitary Surgery—The Role of a CT Scan in Individual Tailoring of Posterior Septum Size Resection

**DOI:** 10.3390/tomography9060172

**Published:** 2023-12-12

**Authors:** Jakub Lubojacký, Lenka Čábalová, Michaela Mladoňová, Viktória Hránková, Tomáš Krejčí, Jakub Mičaník, Maria Miklošová, Lačezar Ličev, Pavel Komínek, Petr Matoušek

**Affiliations:** 1 Department of Otorhinolaryngology and Head and Neck Surgery, Ostrava University Hospital, 70800 Ostrava, Czech Republic; 2 Department of Craniofacial Sciences, Faculty of Medicine, University of Ostrava, 70103 Ostrava, Czech Republic; 3 Department of Anatomy, Faculty of Medicine, University of Ostrava, 70103 Ostrava, Czech Republic; 4 Department of Neurosurgery, Ostrava University Hospital, 70800 Ostrava, Czech Republic; 5 Department of Clinical Neurosciences, Faculty of Medicine, University of Ostrava, 70103 Ostrava, Czech Republic; 6 Department of Radiodiagnostics, Ostrava University Hospital, 70800 Ostrava, Czech Republic; jakub.micanik@fno.cz; 7 Faculty of Electrical Engineering and Computer Science, VŠB Technical University Ostrava, 70800 Ostrava, Czech Republic; lacezar.licev@vsb.cz

**Keywords:** pituitary adenoma, endoscopic transnasal approach, transsphenoidal approach, binostril approach, septal resection

## Abstract

Objective: This study was designed to evaluate the possibility of predicting the minimum size of septal resection for safe tumor extraction in transnasal paraseptal pituitary adenoma resection from preoperative computed tomography scans. Methods: A retrospective CT scan analysis was performed on 20 patients who underwent endoscopic pituitary surgery at the University Hospital in Ostrava. Virtual insertion of the straight instrument into the sphenoid cavity was simulated using a CT scan. The minimum septal resection size was predicted and compared to various diameters in the nasal cavity. The results were then compared with cadaveric dissections, in which septal resections were performed at 1 cm and 2 cm distances from the anterior sphenoid wall. The association between cadaver dissections and CT scan results was studied. Results: A total of 20 patients who underwent endoscopic transnasal surgery for pituitary adenoma between the years 2020 and 2021 were enrolled in the study. The mean virtual posterior septal size resection needed to reach the medial edge of the ICA with the straight instrument, without infracturing the nasal septum, was 13.2 mm. In cadavers with a 1 cm posterior septal resection, the medial edge of the ICA was reached with the straight instrument. In 2 cm resections, it was possible to reach beyond the lateral edge of the ICA. Conclusion: There is no significant correlation between the minimum septal size resection and measured diameters in the nasal cavity. According to our study, a 1 cm resection is sufficient for a non-extended pituitary tumor extraction. More extensive septal resections allow for better maneuverability and overview in the surgical field.

## 1. Introduction

Transnasal endoscopic surgery has emerged as a transformative approach and nowadays is the gold standard in pituitary adenoma treatment [1,2,3]. The hallmark of transnasal endoscopic surgery is its minimally invasive nature. By accessing surgical sites through the nasal passages, this technique eliminates the need for external incisions, reducing trauma to surrounding tissues and resulting in decreased postoperative pain. The use of endoscopes provides surgeons with high-definition, magnified views of the surgical field. This enhanced visualization allows for precise navigation, particularly in anatomically complex areas such as the skull base, sinuses, and upper airway. Patients undergoing transnasal endoscopic surgery often experience reduced morbidity compared to those undergoing traditional open procedures. Shorter hospital stays, quicker recovery times, the absence of external incisions and scars, and a faster return to normal activities contribute to improved overall patient satisfaction [1,2,3,4,5].

While its benefits in terms of minimal invasiveness and improved surgical outcomes are well established, concerns regarding its impact on nasal functions have been raised. For the binostril paraseptal approach, resection of the posterior part of the nasal septum, the anterior wall of the sphenoid cavity, and the intersphenoidal septum are necessary to create access to the sellar region. While a more extensive septal resection means more space, better maneuverability, and an overview of the operating area, it also carries a higher risk of deterioration of nasal functions after surgery. The deterioration of nasal functions leads to a significant loss in the quality of life [4,5,6]. As far as we know, no publications deal with the size of the septal resection. In our study, we attempted to examine whether it is possible to predict the minimum size of a septal resection for safe tumor extraction from a preoperative computed tomography (CT) scan. These findings could be used in the future to tailor the size of the septal resection for each patient individually and minimize the risk of impairing nasal functions after surgery.

## 2. Materials and Methods

For our study, CT scans from both live patients and cadavers were enrolled. Inclusion criteria for patients were pituitary adenoma indicated for transnasal surgery by paraseptal transsphenoidal binostril approach and age between 18 and 70 years. Patients with macroadenomas (tumors larger than 10 mm), previous endonasal surgery, chronic rhinosinusitis, or septal deviation were excluded. Cadavers without previous endonasal surgery or septal deviation were enrolled. 

**Figure 1:**
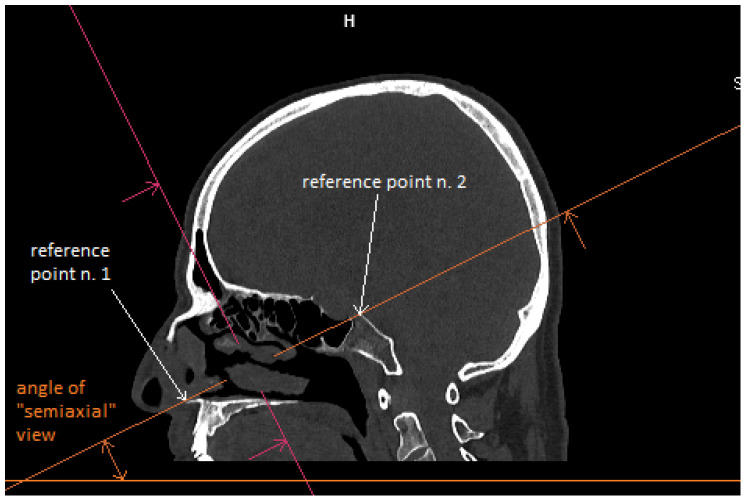
CT scan, sagittal view, creation of semiaxial plane (orange line), reference point n. 1–spina nasalis anterior; reference point n. 2–dorsum sellae.

**Figure 2:**
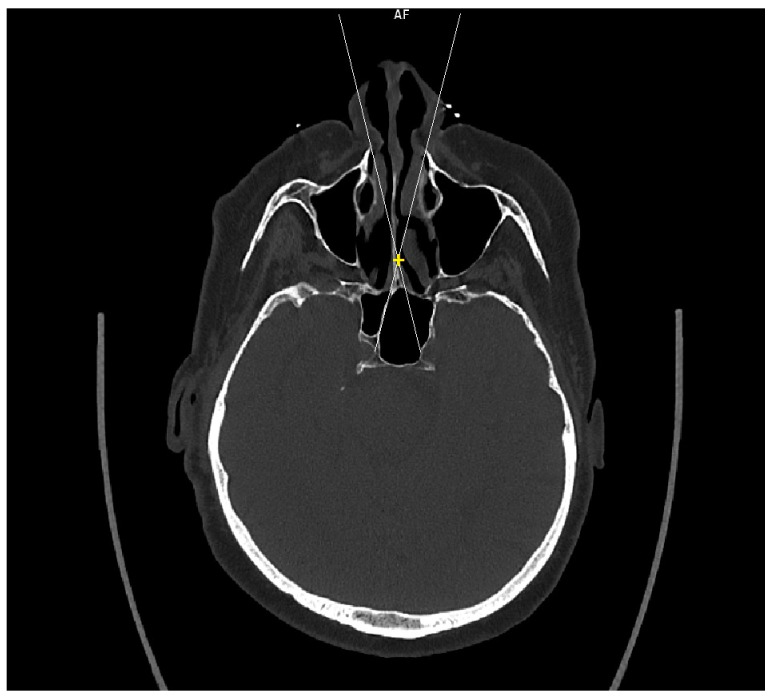
CT scan, semiaxial view, virtual straight instruments inserted into the sphenoid cavity to reach medial edge of the ICAs simulating binostril approach (white lines), crossing of virtual instruments simulating minimal septal resection needed to reach medial edge of the ICAs (yellow cross).

Patient CT scan: Preoperative CT scans from 20 patients with a pituitary adenoma were used for this study. The CT scans were performed on a Siemens Somatom force scanner (Siemens healthineers, Praha, Czech Republic). The measurement was performed with the SyngoVia program, using HrCT images with a slice thickness of 0.6 mm. In each patient, the semi-axial plane was created using multiplanar reconstruction by changing the angle of the axial plane to match the virtual insertion of a straight instrument during surgery through the left nasal cavity into the sphenoid cavity. The defined points for this semi-axial plane were spina nasalis anterior and dorsum sellae (Figure 1). In this semi-axial plane, at the height of the dorsum sellae, the following diameters were measured: the depth of the sphenoid cavity, the distance between the medial edges of the internal carotid arteries (ICA), the width of the pyriform aperture, and the width of the nasal cavity at the level of the attachments of the lower turbinates. The volume of the sphenoid cavity was measured with Fotom software (FOTOM 08 Plus, Ostrava, Czech Republic). Simulation of the virtual insertion of the two straight suctions (one in each nostril) was conducted in a semi-axial view at the height of the dorsum sellae. Instruments were represented with a straight white line; defined points for this white line were the lateral bony edge of the nasal aperture and the medial edge of the ICA. The intersection of the embedded virtual straight instruments (white lines) was marked with a yellow cross. The minimum septal resection required to reach the medial border of the ICA with a straightforward instrument was determined as the distance between the marked yellow cross and the anterior wall of the sphenoid cavity (Figure 2). 

**Figure 3:**
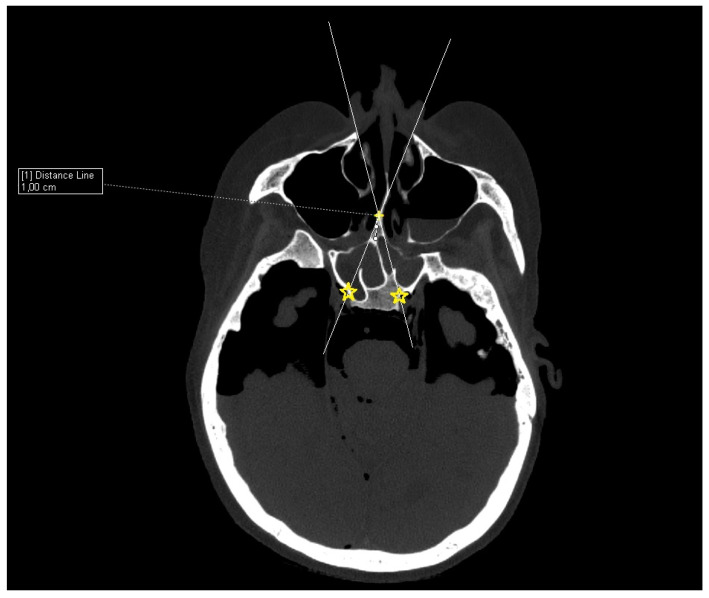
CT scan, semi-axial view, 1 cm distance from anterior sphenoid wall on nasal septum (yellow cross), virtual straight instruments inserted into sphenoid sinus simulating binostril approach (white lines), most lateral area in sphenoid cavity that can be reached with straight instrument with 1 cm septal resection without infracturing the nasal septum (yellow stars).

Cadaver CT scan: Two cadaver heads were used for the CT scan measurement. The CT scans were performed on a Siemens Somatom force scanner (Siemens healthineers, Praha, Czech Republic). With each cadaver head, a different size of septal resection was simulated in SyngoVia software (VB40, Siemens healthineers, Praha, Czech Republic). For the simulation, we used HrCT images with a slice thickness of 0.6 mm in a semi-axial view. A virtual 1 cm posterior part septal resection was performed by marking a yellow cross on the nasal septum 1 cm from the anterior sphenoid wall. A white line was drawn to simulate the insertion of a straight instrument. The defined points for this line were the bony lateral part of the nasal aperture and the marked yellow cross on the nasal septum. The place where this ongoing white line crossed the posterior bony edge of the sphenoidal sinus represented the most lateral point that could be reached in the sphenoid sinus with a straight instrument without infracturing the nasal septum. This point was then marked with a yellow star. The same procedure was performed for both nostrils. The same principle and procedure were used for a 2 cm resection simulation with the second cadaver head (Figure 3).

**Figure 4:**
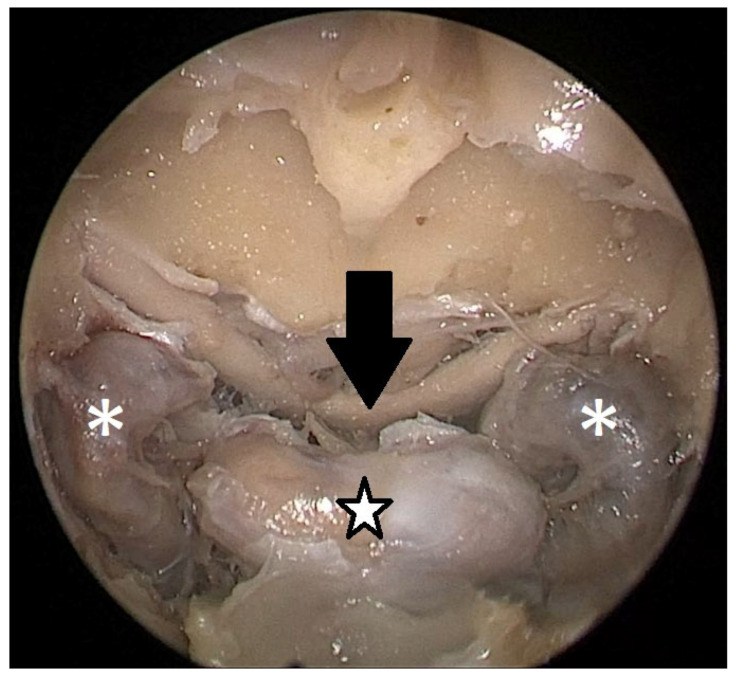
Opened sphenoidal sinus, endoscopic view, fully exposed ICAs (white asterisks), pituitary gland (black and white star), and optical chiasm (black arrow).

**Figure 5:**
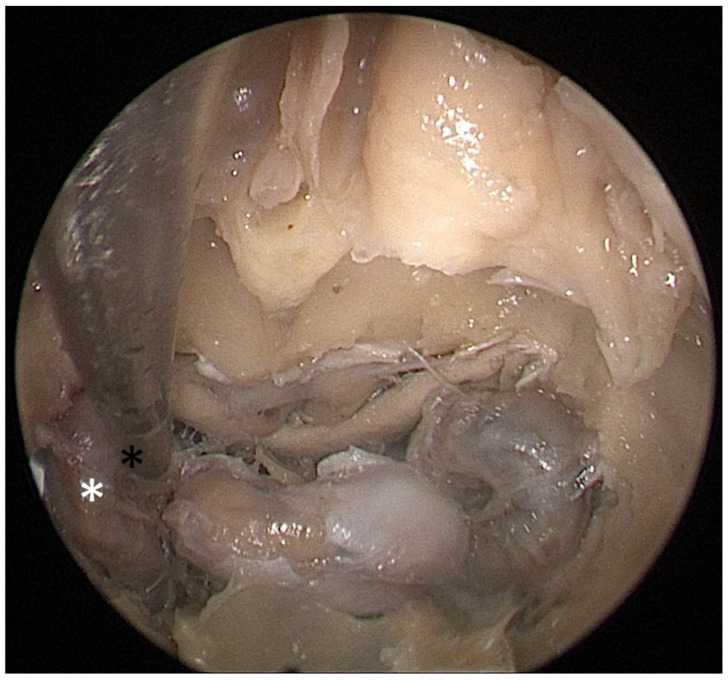
Opened sphenoidal sinus, endoscopic view, straight suction (black asterisk) reaching the medial border of ICA (white asterisk) with a 1 cm septal resection.

**Figure 6:**
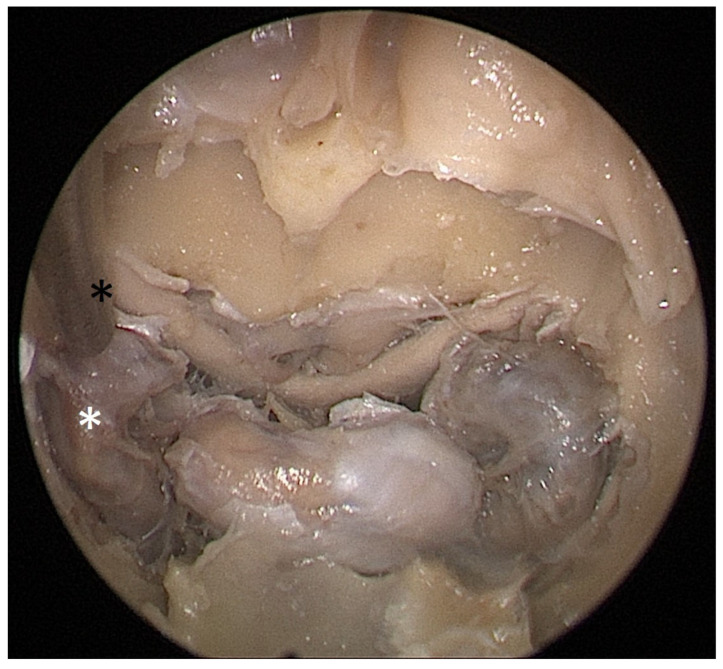
Opened sphenoidal sinus, endoscopic view, straight suction (black asterisk) reaching lateral margin of ICA (white asterisk) with 2 cm septal resection.

Cadaver pituitary surgery: On each of the two cadaveric heads, we used the paraseptal binostril approach for the sphenoid sinus. A 30° Storz endoscope (Karl-Storz, Austria, Vienna) was used. The head was mounted in a Mayfield cranial stabilization device. The surgery started with a lateralization of the inferior and medial turbinates. The anterior sphenoid wall and ostium to sphenoid sinus were identified. Using the straight suction with a marked scale, 1 cm and 2 cm distances from the anterior sphenoid wall were measured and marked with a scalpel on the posterior part of the septum. Using a scalpel, Kerrison punch, and backbiter punch, a posterior septal resection 1 cm from the anterior wall of the sphenoid was performed. We continued with the wide opening of the sphenoid cavity and resection of the intersphenoidal septum and the full exposure of the posterior wall, both ICAs, and optocarotic recesses (OCR). The posterior wall of the sphenoid sinus and dura were resected to better identify anatomic landmarks, with both ICAs, pituitary gland, chiasma, and optic nerves visible at the end of a resection (Figure 4). The straight suction was inserted into the sphenoid sinus so that the instrument’s tip was in the most lateral position in the sphenoid sinus without infracturing the rest of the nasal septum. A picture was taken in this position to evaluate the most lateral part of the sphenoid sinus that could be reached with a straight instrument with a 1 cm septal resection without infracturing the nasal septum (Figure 5). Enlargement of the septal resection to 2 cm from the anterior sphenoid wall was performed with a backbiter punch and scalpel. The straight suction was inserted into the sphenoid sinus so that the instrument’s tip was in the most lateral position in the sphenoid sinus without infracturing the rest of the nasal septum. A picture was taken in this position to evaluate the most lateral part of the sphenoid sinus that could be reached with a straight instrument, with a 2 cm septal resection, without infracturing the nasal septum (Figure 6).

Both septal size resections were then compared regarding maneuverability and overview of the operated area (subjectively evaluated by the surgeon; 1—bad, 2—normal, 3—good).

The Shapiro–Wilk normality test was used to determine the normality of the demography data. Other parameters were calculated by using Fisher’s exact test for count data. All methods were used from R-project libraries (R Core Team (2020)).

## 3. Results

In total, 20 patients who underwent endoscopic transnasal pituitary surgery with the paraseptal transsphenoidal binostril approach between 2020 and 2021 were enrolled in the study. There were 14 men and 6 women. The mean age was 60 years. 

The mean intercarotid distance was 17.2 mm (ranging from 12 to 23.8 mm). The mean depth of the sphenoid sinus was 27.8 mm (ranging from 17.3 to 39.8 mm). The mean volume of the sphenoid sinus was 10.2 mL (ranging from 4.11 to 14.2 mL). The mean width of the pyriform aperture was 23.9 mm (ranging from 21.4 to 26.7 mm). The mean width of the nasal cavity at the height of the attachment of the lower turbinate was 21.4 mm (ranging from 14.8 to 26.6 mm). The mean size of the septal resection theoretically needed to reach the medial edge of the ICA with a straight instrument, without infracturing the nasal septum, was 13.2 mm (ranging from 4.9 to 18.2 mm). Results are summarized in Table 1.

No significant relationship between the minimum septal resection distance and depth of the sphenoid sinus (*p* = 0.7) was found. No statistically significant correlation, when comparing the minimum septal resection size with the intracarotid distance (*p* = 0.09), nor with the volume of the sphenoid cavity (*p* = 0.14), was found. There was no significant relationship between the minimum septal resection size and the width of the nasal cavity at the attachment of the lower turbinates (*p* = 0.66), nor the width of the pyriform aperture (*p* = 0.45).

According to the CT scan measurement on the cadaveric heads, a 1 cm resection of the posterior part of the nasal septum was enough to reach the medial edge of the ICA with a straight instrument, while a 2 cm resection was enough to reach the lateral edge of the ICA. Transnasal endoscopic surgery of the cadaveric heads proved the CT scan findings to be true. A 1 cm resection of the posterior part of the nasal septum was enough for the surgeon to reach the medial side of the ICA without infracturing the nasal septum with the straight instrument. A 2 cm resection was enough for the surgeon to reach the lateral edge of the ICA without infracturing the nasal septum. An approach with a 1 cm resection is sufficient for safe tumor extraction in non-extended cases. The same results were observed in both cadaveric heads. Subjective evaluation of the maneuverability and overview of the operated area was better with a 2 cm resection.

## 4. Discussion

With its intricate anatomy and multifaceted functions, the human nose is a remarkable organ that plays a vital role in our daily lives. Beyond its role in breathing, the nose is responsible for our sense of smell, enhances our sense of taste, contributes to speech and phonation, and is a crucial component of our immune defense system [1,3,6].

The sense of smell, or olfaction, is a pivotal component of human perception, offering valuable insights into the surrounding environment. This sensory system relies on the intricate interaction between specialized olfactory receptors and odor molecules. Olfactory receptors are found within the nasal mucosa around the lamina cribrosa, superior turbinate, and posterior part of nasal septum. Receptors belong to the family of G-protein-coupled receptors. These receptors are remarkably diverse, allowing for detecting a vast array of odorants. The olfactory bulb plays a crucial role in processing and interpreting these signals. Here, odor discrimination and identification occur, enabling individuals to distinguish between countless scents [4,5]. The significance of olfaction extends beyond mere odor perception. It influences taste perception, aids in detecting danger through noxious odor recognition, affects our mood, and even contributes to social interactions through detecting pheromones and subtle emotional cues conveyed by scent [5,6,7,8,9]. The brain centers mediating the sense of smell functionally overlap with centers for processing emotions, moods, and memory formation. Therefore, the loss of smell is associated with an increased susceptibility to depression [4,5,6,7,9].

Deterioration in nasal airflow, whether due to structural issues, edema, or other factors, can lead to snoring and difficulty breathing, especially during sleep. This can impact sleep quality and overall energy levels and can also lead to cardiovascular diseases. Chronic congestion also often leads to headaches and facial pain. Nasal issues, especially those that affect appearance, such as persistent sniffling or frequent nose blowing, can be socially challenging. Breathing difficulties and discomfort associated with nasal issues may limit an individual’s ability to engage in physical activities. This can contribute to a sedentary lifestyle, impacting overall physical health and well-being [1,5,7,9].

Endoscopic transnasal paraseptal transsphenoidal approaches are the gold standard for the surgical therapy of pituitary pathologies [1,3,8,9]. This approach is minimally invasive, involving access to the pituitary gland through the natural nasal passages, which avoids the need for extensive skull or facial incisions and reduces trauma to surrounding tissues. It also offers shorter hospital stays with faster recovery rates. From surgeons’ perspectives, it involves fewer manipulations of the brain tissue, reducing the risk of brain injury and complications associated with open surgery. The endoscope used in transnasal endoscopic pituitary surgery (TEPS) provides superior visualization and illumination of the surgical field, enabling precise tumor removal and minimizing damage to surrounding healthy tissues [3,6,8]. 

On the other side, in most patients, the surgery is performed in a healthy nasal cavity. Access to the pituitary gland must compromise between creating sufficient space for tumor extraction and preserving nasal anatomical structures. Disruption of the normal nasal anatomy can lead to the postoperative deterioration of the nasal functions, especially the sense of smell [10,11,12,13,14,15]. 

In the early weeks after surgery, the deterioration of the nasal functions can be caused mainly by swelling and the formation of crusts in the operated area, which prevent proper airflow [13,14,15,16,17]. With a longer passage of time after the operation, the anatomical changes in the nasal cavity and the associated airflow changes can cause nasal function deterioration [11,13,14,15,18]. In a healthy nose, air flows laminarly. Laminar airflow allows the nasal mucosa and cilia to effectively filter out particles, dust, allergens, and pathogens in the inhaled air. It is also vital for proper humidification and temperature regulation, as the slow, laminar flow allows the air to come into contact with the nasal mucosa. 

According to Eccles et al., the posterior part of the nasal septum, which is resected during surgery, is essential for maintaining laminar airflow in the olfactory area. The disruption of this flow leads to the formation of turbulent currents, obstruction of airflow, reduction in air humidity, and drying of the mucous membrane. These factors may cause crusting formation and olfaction to worsen postoperatively [14,19,20].

Some of the possible complications associated with transnasal skull base surgery are mentioned below. Proper postoperative care is vital for accurate healing.

One of the primary concerns after transnasal pituitary surgery is the potential for cerebrospinal fluid leakage (CSF). This can occur when the protective layer surrounding the brain is breached during surgery. Postoperatively, patients are monitored closely for signs such as clear nasal drainage, headaches, or signs of an infection. Prompt identification and intervention, including lumbar drains and surgical sealants, are crucial for managing this complication. CSF can also contribute to the development of meningitis, a potentially severe inflammation of the membranes surrounding the brain and spinal cord. The early recognition of symptoms such as fever, neck stiffness, nasal discharge, facial pain, and altered mental status is crucial for timely intervention and effective management. Vigilant nasal hygiene and prophylactic antibiotics are crucial in minimizing this risk. 

While transnasal skull base surgery is designed to minimize bleeding, hemorrhage remains a potential complication. One of the most fearful complications is injury to the internal carotid artery. The proximity of the carotid artery to the skull base poses a unique challenge during surgery. Accidental injury to the carotid artery can lead to severe bleeding, putting the patient at risk of stroke and other life-threatening complications. The intricate anatomy and limited visualization in this surgical approach contribute to the complexity of managing such incidents. While carotid artery injuries during transnasal endoscopic skull base surgery are rare, awareness of the potential risk factors is crucial. Anatomical variations, tumor characteristics, and surgeon experience can all influence the likelihood of encountering the carotid artery during the procedure. The prompt and effective management of carotid artery injury is paramount. Surgeons must be prepared to employ various techniques, including direct repair with muscle patches or endovascular interventions. The decision making process depends on the extent of the injury and the patient’s overall condition. The literature suggests that outcomes following carotid artery injury during transnasal endoscopic skull base surgery are variable. Successful management often requires a multidisciplinary approach involving neurosurgeons, vascular surgeons, and interventional radiologists. However, despite optimal interventions, complications such as neurological deficits, postoperative morbidity, or even death may occur. After surgery, the close monitoring of postoperative bleeding, along with immediate medical attention in case of severe bleeding, is vital [15,17,19,20].

For better healing of the nasal mucosa, saline irrigations are recommended; they clean the nasal cavity from crusts and coagulums and prevent postoperative synechia formation. Nasal irrigation is often recommended multiple times daily for at least a few weeks after surgery. The recommended volume of saline solution per irrigation can vary but is usually in the range of 240 mL to 350 mL. To enhance comfort, the saline solution should be at a lukewarm or body temperature. Before dismissal to home care, an endoscopic examination with crust removal is recommended [15,17].

Recent studies suggest that TEPS is generally associated with stable or only mildly deteriorated nasal functions and a low risk of complications. Transient olfactory disturbances may occur immediately after surgery; these typically resolve within a few weeks to months. Long-term significant deterioration in olfaction is uncommon. The prevalence of permanent postoperative anosmia after TEPS is under 5%. Factors such as the type and size of pituitary lesions, the extent of surgical resection, and the surgical team’s experience could influence olfactory outcomes [17,18,19,20,21].

The relationship between the size of the septal resection and the postoperative nasal functions has not been described. At the same time, no articles in the literature describe the size of the septal resection and its effect on the clarity of the operative field, maneuverability, or radicality of the operation. The standard procedure is to resect the posterior part of the septum in such a way that the tuberculum sellae and the bilateral cavernous internal carotid artery (c-ICA) are exposed without measuring the size of the septum resection [19,20,21].

In our study, we used preoperative CT scans from 20 patients with a pituitary adenoma to measure the minimum septal resection that would be sufficient to fully expose and reach the tuberculum sellae and both c-ICA with a straight instrument. These findings were then verified on two cadaveric heads. 

We discovered that the mean minimum resection of the posterior septum, measured from the anterior wall of the sphenoid sinus, was 13.2 mm. This distance was, however, very individual and varied from 4.9 mm to 18.2 mm. There was no correlation found between the measured proportions (intercarotid distance, width of the pyriform aperture, width of the nasal cavity at the level of the attachment of the lower turbinate, depth of the sphenoidal sinus, and volume of the sphenoidal sinus) and the size of the minimum needed septal resection, which was very surprising. It is necessary to consider other factors, such as tissue resistance, the size of the turbinates, or the volume of the nasal cavity and paranasal sinuses, to fully understand the complex relationship between the minimum necessary septum resection and nasal anatomy. 

Another part of our study involved cadavers. Using a CT scan, two cadaveric heads were measured; a 1 cm resection was enough to reach the medial part of the ICA with a straight instrument, and 2 cm allowed us to reach the lateral margin of the ICA. To verify these data, pituitary surgery was performed on cadaveric heads.

Each head was operated on twice. First, a binostril approach and a 1 cm posterior septal resection were performed. This allowed us, in both cadavers, to comfortably reach the medial part of the carotids bilaterally with straight suction. This resection size would be sufficient for a safe tumor extraction in non-extended tumors. Then, a 2 cm extension of the septal resection was performed. A more extensive resection allowed us to reach the lateral margin of the ICA bilaterally. It also gave us better maneuverability and an overview of the operated area.

The most significant limitation of CT measurement is the impossibility of considering the tissues’ flexibility and resistance. During the surgery, we can create more space by increasing the instrument’s pressure on the nasal structures without damaging or infracturing them. This cannot be simulated with a CT measurement.

Due to the enormous variability of the paranasal sinuses, it is also challenging to find reference points that would be both accurate and consistent and, at the same time, correspond as closely as possible to the reality of inserting the instrument into the nasal cavity during actual surgery.

## 5. Conclusions

According to our study, there is no significant correlation between the minimum septal size resection and the intercarotid distance, width of the pyriform aperture, width of the nasal cavity at the level of the attachment of the lower turbinate, depth of the sphenoidal sinus, or volume of sphenoidal sinus that could help us predict the minimum size of septal resection before surgery using a CT scan. Pituitary surgery on cadavers, as well as CT scans of the cadaver heads, proved that a 1 cm resection was enough to reach the medial edge of the ICA. A 2 cm resection allowed us to reach the lateral edge of the ICA. A smaller 1 cm resection is sufficient for non-extended pituitary tumor extraction. More extensive septal resection allows better maneuverability and overview of the operated area. Further studies comparing nasal functions in different septal size resections are needed to fully understand whether the more significant risk of impairing nasal functions accompanies bigger resections.

## Figures and Tables

**Table 1:** Summary of measured distances in nasal cavity.

	Minimum Value	Maximum Value	Mean Value
Intercarotid distance	17.2 mm	12 mm	23.8 mm
Depth of sphenoid sinus	27.8 mm	17.3 mm	39.8 mm
Volume of sphenoid sinus	10.2 mL	4.11 mL	14.2 mL
Width of pyriform aperture	23.9 mm	21.4 mm	26.7 mm
Width of the nasal cavity *	21.4 mm	14.8 mm	26.6 mm
Size of septum resection **	13.2 mm	4.9 mm	18.2 mm

* width of the nasal cavity at the height of the attachment of the lower turbinate, ** minimal size of septum resection needed to reach medial edge of the ICA with straight instrument without infracturing nasal septum.

## Data Availability

Restrictions apply to the availability of these data. Data generated or analyzed during this study are available from the corresponding author by request, subject to institutional review and a data use agreement.
